# Sex‐ and Age Group‐Specific Fracture Incidence Rates Trends for Type 1 and 2 Diabetes Mellitus

**DOI:** 10.1002/jbm4.10836

**Published:** 2023-10-27

**Authors:** Mohamad I Nasser, Annika Vestergaard Kvist, Peter Vestergaard, Richard Eastell, Andrea M Burden, Morten Frost

**Affiliations:** ^1^ Department of Endocrinology and Metabolism, Molecular Endocrinology Stem Cell Research Unit (KMEB) Odense University Hospital Odense Denmark; ^2^ Department of Clinical Research University of Southern Denmark Odense Denmark; ^3^ Steno Diabetes Center Odense Odense University Hospital Odense Denmark; ^4^ Pharmacoepidemiology Group, Institute of Pharmaceutical Sciences ETH Zurich Zurich Switzerland; ^5^ Steno Diabetes Center North Denmark Aalborg University Hospital Aalborg Denmark; ^6^ Department of Endocrinology Aalborg University Hospital Aalborg Denmark; ^7^ Department of Clinical Medicine Aalborg University Hospital Aalborg Denmark; ^8^ Academic Unit of Bone Metabolism University of Sheffield Sheffield UK; ^9^ Mellanby Centre for Musculoskeletal Research University of Sheffield Sheffield UK; ^10^ Leslie Dan Faculty of Pharmacy University of Toronto Toronto Canada

**Keywords:** FRACTURES, INCIDENCE, TRENDS, TYPE 1 DIABETES MELLITUS, TYPE 2 DIABETES MELLITUS

## Abstract

The incidence of major osteoporotic fractures has declined in men and women in Western countries over the last two decades. Although fracture risk is higher in persons with diabetes mellitus, trends of fractures remain unknown in men and women with diabetes. We investigated the trends in fracture incidence rates (IRs) in men and women with type 1 diabetes mellitus (T1D) and type 2 diabetes mellitus (T2D) in Denmark between 1997 and 2017. We identified men and women aged 18+ years who sustained a fracture (excluding skull and facial fractures) between 1997 and 2017 using the Danish National Patient Registry. We calculated sex‐specific IRs of fractures per 10,000 person‐years separately in persons with T1D, T2D, or without diabetes. Furthermore, we compared median IRs of the first 5 years (1997–2002) to the median IRs of the last 5 years (2012–2017). We identified 1,235,628 persons with fractures including 4863 (43.6% women) with T1D, 65,366 (57.5% women) with T2D, and 1,165,399 (54.1% women) without diabetes. The median IRs of fractures declined 20.2%, 19.9%, and 7.8% in men with T1D, T2D, and without diabetes, respectively (*p*‐trend <0.05). The median IRs decreased 6.4% in women with T1D (*p*‐trend = 0.35) and 25.6% in women with T2D (*p*‐trend <0.05) but increased 2.3% in women without diabetes (*p*‐trend = 0.08). Fracture IRs decreased in men with both diabetes types and only in women with T2D, highlighting the need for further attention behind the stable trend observed in women with T1D. © 2023 The Authors. *JBMR Plus* published by Wiley Periodicals LLC on behalf of American Society for Bone and Mineral Research.

## Graphical abstract

The trends in incidence rates of fractures declined in men with type 1 and 2 diabetes mellitus and in women with type 2 diabetes mellitus in Denmark between 1997 and 2018. The decline may be attributable to improved diabetes management and increased awareness of bone complications in diabetes. Further investigation is required for the stable trend observed in incidence rates of fractures for women with type 1 diabetes mellitus.

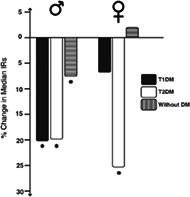

## Introduction

Fractures are a burden to both patients and the health care system by limiting functionality and quality of life and increasing morbidity and mortality.^(^ ^1^, ^2^, ^3^, ^4^ ^)^ Both type 1 diabetes mellitus (T1D) and type 2 diabetes mellitus (T2D) have been identified as important risk factors for fractures.^(^ ^5^, ^6^, ^7^, ^8^ ^)^ In a recent meta‐analysis,^(^ ^9^ ^)^ hip fractures were 4.9‐fold and 1.9‐fold more common in persons with T1D and T2D, respectively. Similarly, a higher risk of incident vertebral fractures was reported in persons with T2D compared with persons without diabetes.^(^ ^10^ ^)^ Although young and middle‐aged adults with T1D have low bone mineral density (BMD) and an increased fracture risk,^(^ ^6^, ^7^ ^)^ persons with T2D have a normal or even an increased BMD^(^ ^7^, ^11^ ^)^ but increased cortical porosity.^(^ ^12^ ^)^

The pathogenesis of skeletal fragility in T1D and T2D is considered multifactorial and encompasses shared risk factors, including hyperglycemia that decreases bone cell activity and the accumulation of advanced glycation end products in the bone matrix, which decreases bone material properties.^(^ ^13^ ^)^ In addition, persons with T1D and T2D have higher risk of falls,^(^ ^14^ ^)^ which could be attributable in part to other comorbidities such as atherosclerosis affecting sensory‐motor reflexes or glucose‐lowering medication use.^(^ ^15^ ^)^ For instance, insulin or sulfonylureas are associated with a higher falls risk because of drug‐induced hypoglycemia^(^ ^16^, ^17^ ^)^ and in turn a higher fracture risk, unlike glucagon‐like peptide‐1 receptor agonists (GLP‐1), which have a neutral effect on fracture risk.^(^ ^18^ ^)^

Previous studies have identified that the incidence of major osteoporotic fractures in the general population has declined in Nordic and Western countries over the last two decades.^(^ ^19^, ^20^, ^21^ ^)^ Furthermore, this decline in incidence rates (IRs), specifically in hip fractures for patients ≥50 years, has been observed in registry data from several countries around the world.^(^ ^22^ ^)^ In Denmark, the age‐specific IRs of hip fractures declined between 2005 and 2015 by 30% in 50+‐year‐old adults.^(^ ^19^ ^)^ Furthermore, a 31% and 19% decline in hip fracture rates was observed in 50+‐year‐old Danish women and men, respectively, between 1995 and 2010.^(^ ^20^ ^)^

Given the emergence of novel glucose‐lowering treatment modalities associated with less fracture risk and the improved clinical management of T1D and T2D, we hypothesize that the declining trend in IRs of fractures in the general population^(^ ^23^ ^)^ is also found in men and women with T1D and T2D. Therefore, our primary aim in this study was to investigate if the trends in IRs of fractures have also declined in men and women with T1D and T2D. Also, considering the debut of T1D early in life, fracture risk could increase at a lower age in persons with T1D than observed in persons with T2D or without diabetes. Thus, we also assessed trends in the IRs of fractures in persons with T1D and T2D in different age groups.

## Subjects and Methods

### Data sources

This observational study is based on data from the Danish National Patient Register (DNPR), which covers all inpatient contacts from 1977 onward. In addition, the DNPR includes information on all outpatient contacts to hospitals, outpatient clinics, and emergency room visits since 1995. The International Classification of Diseases 8th edition (ICD‐8) was used to code for diagnosis of diseases before 1994, and the International Classification of Diseases 10th edition (ICD‐10) has been used since 1994. Denmark has a universal coverage of health care that is free of charge. Previous studies have demonstrated high validity for fracture codes in this database.^(^ ^24^ ^)^ To identify medications used before a fracture, we used the Danish Medicines Agency Register of Medicinal Products Statistics (RMPS), which is a nationwide prescription database for all the prescriptions dispensed since 1995 at community pharmacies. The medications are classified by their Anatomical Therapeutic Chemical (ATC) codes, and information is available on the dates of prescriptions and the dosages. In Denmark, health care data are linked using a personal 10‐digit code (CPR) social security number assigned to all Danish residents.

### Study design and study population

We identified all patients aged 18 years or older with one or more fractures between 1997 and 2017. We used ICD‐10 codes to identify eligible bone fractures, except for facial and cranial fractures (eligible fractures: S22‐S92, T02, T08, T10, T12, T142, M484, M485, M80, M843, M844). We applied a washout period of 365 days to avoid double counting of fractures. Therefore, we identified the first fracture code for all the eligible fractures from 1997 onward and assessed if a person had a previous code for the same fracture site in the prior 365 days.

Persons with T1D were identified with ICD‐8 code 249 or ICD‐10 code E10 in addition to at least one fulfilled prescription of insulin or an insulin analog and the absence of T2D‐prescribed medications (ATC codes for T1D medications A10A, except A10AE54 and A10AE56). Persons with T2D were identified with ICD‐8 code 250 or ICD‐10 codes E11, E12, E13, or E14, or at least one fulfilled prescription of glucose‐lowering medications (A10A or A10B). We excluded women with polycystic ovary syndrome (PCOS), defined as being prescribed metformin together with clomiphene (ATC codes A10BA02 and G03GB02), being prescribed metformin together with antiandrogens in combination with estrogen (ATC codes A10BA02 and G03HB), or being prescribed metformin and who had a PCOS diagnosis (ATC code A10BA02 and ICD‐10 code E282). We identified T1D, T2D, or without diabetes status before each fracture, as a person can be without diabetes at the time of the first fracture but could potentially develop diabetes before a later fracture.

### Statistical analysis

We summarized the baseline characteristics of the study population at the time of the first eligible fracture between 1997 and 2017 and stratified the data by diabetes type into T1D, T2D, or without diabetes. We assessed the use of medications before the first fracture. Means and standard deviations (SD) were used for continuous data, while counts and proportions were used for categorical data. Subsequently, we calculated the annual incidence of fractures in the study population (number of fractures/10,000 person‐years [py]), and the corresponding 95% confidence intervals (CIs). The number of eligible fractures per calendar year was divided by the total number of persons alive for each of the three groups (T1D, T2D, and without diabetes) in the same calendar year as identified from Statistics Denmark (https://www.dst.dk/en/Statistik/emner/borgere/befolkning/befolkningstal, FOLK1A), and a database incorporating all persons with diabetes in Denmark from 1977 to 2017 (project 703382 in Statistics Denmark). Age‐specific IRs in seven age groups (18–29, 30–39, 40–49, 50–59, 60–69, 70–79, and 80+ years) were calculated. In addition, the median IR for the first 5 years (1997–2001) was compared with the median IR for the last 5 years (2013–2017). Furthermore, linear regression models were used to investigate if changes in IRs over time were statistically significant, and pairwise *t* tests were conducted to compare the trends among groups. Additionally, considering the importance of understanding the adoption of newer glucose‐lowering medications as a measure of progress in diabetes management, we described the trends of glucose‐lowering medication use over the study period. Furthermore, as there are differences in mean age and the proportion of men to women across the study groups, we ran a sensitivity analysis to describe the sex‐ and age‐adjusted trends in IRs of fractures among persons with T1D, T2D, and without diabetes (Supplemental Fig. S1). The statistical analyses were performed using SAS Enterprise (version 7.15) and RStudio (version 4.0.3).

## Results

### Cohort characteristics

**Fig. 1:**
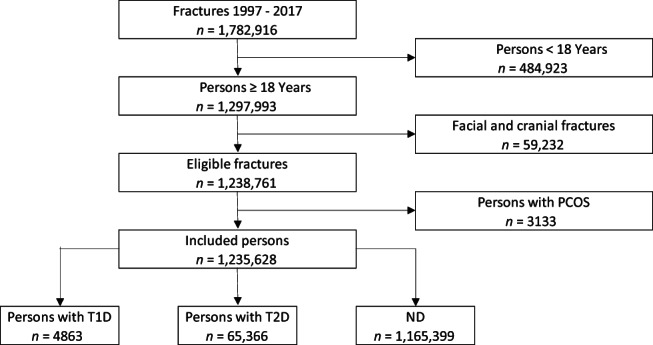
Flow diagram of patients eligibility. *n* = number of patients; ND = without diabetes; PCOS = polycystic ovary syndrome; T1D = type 1 diabetes mellitus; T2D = type 2 diabetes mellitus.

We identified 1,782,916 persons with at least one fracture between 1997 and 2017 from the DNPR database (Fig. 1). We excluded persons younger than 18 years (*n* = 484,923), persons with facial and cranial fractures (*n* = 59,232), and women suspected to have PCOS (*n* = 3133). A total of 1,235,628 persons were included in this study. Persons were stratified according to their diabetes type, into persons with T1D (*n* = 4863), persons with T2D (*n* = 65,366), or persons without diabetes (*n* = 1,165,399).

We summarized the baseline characteristics of the study population in Table 1, stratified by the diabetes type. The mean age of persons with T1D was 49.3 years (SD = 19.9), 68.6 (SD = 14.5) in persons with T2D, and 52.9 (SD = 21.5) in persons without diabetes. In T1D, fractures were more common in men (56.3%) than in women, yet, in T2D and without diabetes, fractures were more common in women (57.5% and 54.1%, respectively). Fractures were more common at a younger age (18 to 29 years) in persons with T1D and persons without diabetes (Table 1). By contrast, fractures were less prevalent in the younger age group (18 to 29 years) than in older age groups in persons with T2D. Overall, for those aged <60 years, fractures were more prevalent in persons with T1D than in persons with T2D. However, for adults aged 60+ years, fractures were more prevalent in persons with T2D than in persons with T1D.

**Table 1:** Baseline Characteristics of the Study Population at First Fracture, Stratified by Diabetes Type into Type 1 Diabetes Mellitus (T1D), Type 2 Diabetes Mellitus (T2D), or Without Diabetes

	T1D	T2D	Without diabetes
Total patients	*n* = 4863	*n* = 65,366	*n* = 1,165,399
Sex, *n* (%)			
Men	2740 (56.3)	27,761 (42.4)	533,895 (45.8)
Women	2123 (43.6)	37,605 (57.5)	631,504 (54.1)
Age (years), mean (SD)	49.3 (19.9)	68.6 (14.5)	52.9 (21.5)
Age categories (years), *n* (%)			
18–29	1036 (21.3)	746 (1.1)	221,224 (18.9)
30–39	691 (14.2)	1753 (2.6)	146,120 (12.5)
40–49	734 (15.0)	4182 (6.4)	155,075 (13.3)
50–59	797 (16.3)	9531 (14.5)	178,573 (15.3)
60–69	672 (13.8)	14,580 (22.3)	156,752 (13.4)
70–79	547 (11.2)	17,472 (26.7)	144,538 (12.4)
80+	386 (7.9)	17,102 (26.7)	163,117 (14.0)
Diabetes duration (years), mean (SD)	9.1 (7.5)	6.8 (8.1)	NA
Retinopathy, *n* (%)	1602 (32.9)	11,577 (17.7)	6030 (0.5)
Nephropathy, *n* (%)	386 (7.9)	5031 (7.7)	1274 (0.1)
Neuropathy, *n* (%)	719 (14.8)	9582 (14.6)	10,616 (0.9)
Glucose‐lowering medications, *n* (%)			
Biguanides	NA	35,153 (53.8)	524 (0)
Sulfonylureas	NA	34,490 (52.8)	533 (0)
Thiazolidinediones	NA	782 (1.2)	<5 (0)
DPP‐4 inhibitors	NA	3016 (4.6)	<5 (0)
Insulin and analogues	4812 (99)	21,274 (32.5)	43 (0)
GLP‐1 agonists	NA	2139 (3.3)	12 (0)
SGLT2 inhibitors	NA	453 (0.7)	NA

DPP‐4 inhibitors = dipeptidyl peptidase‐4 inhibitors; GLP‐1 = glucagon‐like peptide‐1 agonists; NA = not applicable; SD = standard deviation; SGLT2 = sodium‐glucose cotransporter‐2 inhibitors; T1D = type 1 diabetes mellitus; T2D = type 2 diabetes mellitus.

Retinopathy was more common in persons with T1D compared with persons with T2D (32.9% and 17.7%, respectively), but the prevalence of neuropathy (7.9% and 7.6% in T1D and T2D, respectively) and nephropathy (14.7% and 14.6% in T1D and T2D, respectively) was almost similar between the two groups. Persons with T2D were mainly prescribed oral glucose‐lowering medications including biguanides (53.8%) and sulfonylureas (52.8%). In addition, 32.5% of persons with T2D were prescribed insulin or analogues. In contrast, dipeptidyl peptidase 4 inhibitors (DPP‐4 inhibitors), GLP‐1 agonists, sodium‐glucose cotransporter‐2 inhibitors (SGLT2), and thiazolidinediones were prescribed less in persons with T2D (4.6%, 3.3%, 0.7%, and 1.2%, respectively).

### IRs by sex

**Fig. 2:**
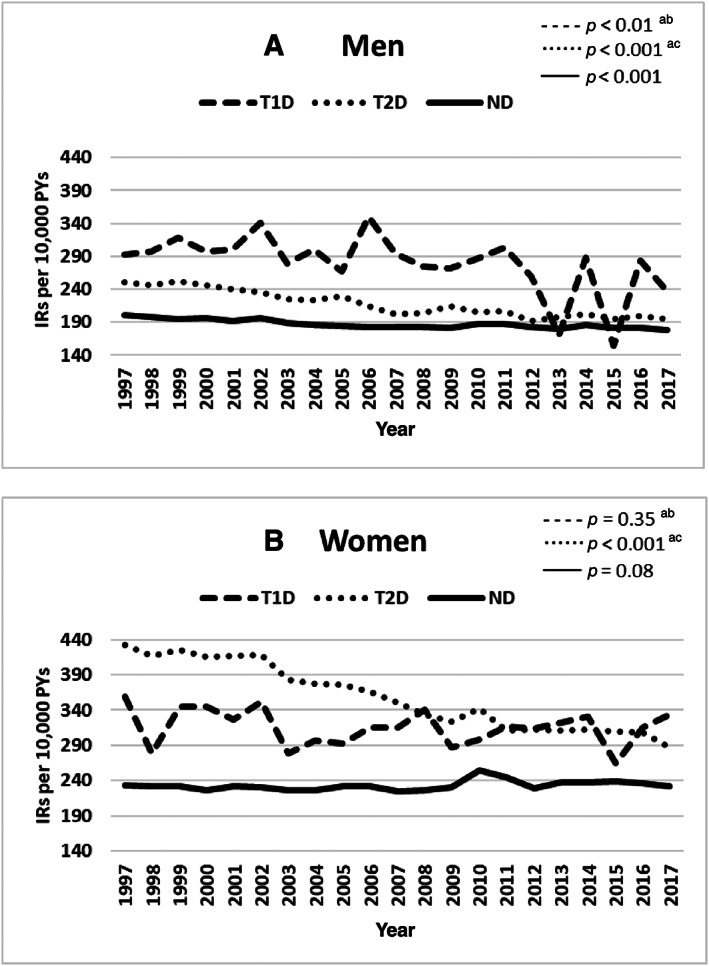
Incidence rates of fractures in Danish adults aged 18 years or older, stratified by diabetes mellitus type and sex (*A*, *B*) according to calendar year (1997–2017). The trends in the slope for each group were evaluated using linear regression, and we used pairwise *t* tests to compare the trends among groups. Symbols for the pairwise *t* test: ^a^ *p* < 0.05 for T1D versus T2D, ^b^ *p* < 0.05 for T1D versus ND, and ^c^ *p* < 0.05 for T2D versus ND. IR = incidence rate; ND = without diabetes; PYs = person‐years; T1D = type 1 diabetes mellitus; T2D = type 2 diabetes mellitus.

Changes in IRs of fractures were most prominent in men with T1D and T2D (Fig. 2*A*) and in women with T2D (Fig. 2*B*). The annual counts, IRs, and 95% CIs in men and women, stratified by diabetes status, are provided in Supplemental Tables S1 and S2.^(^ ^25^ ^)^ The median IR of fractures decreased 19.9% and 25.6% in men and women with T2D, respectively (Table 2). Similarly, it decreased 20.2% and 6.4% in men and women with T1D, respectively (Table 2). Although the median IR of fractures increased 2.3% in women without diabetes, it decreased 7.8% in men without diabetes (Table 2). Regression analyses showed declining trends in men in all groups (*p*‐trend <0.01 in T1D, *p*‐trend <0.001 in T2D and without diabetes) (Fig. 2*A*), and only in women with T2D (*p*‐trend <0.001) (Fig. 2*B*). We identified statistically significant differences in trends between persons with T1D, persons with T2D, and persons without diabetes in men and in women (*p* < 0.05) (Fig. 2*A*, *B*).

**Table 2:** Median IRs per 10,000 Person‐Years With the Median Change in Percentage for Persons With T1D, Persons With T2D, and Persons Without Diabetes, Stratified by Sex and Age (in Years)

	T1D			T2D			Without diabetes		
	Median IR/PYs	Change (%)	Increase/decrease	Median IR/PYs	Change (%)	Increase/decrease	Median IR/PYs	Change (%)	Increase/decrease
Total									
1997–2001	317.3	6.1	↓	329.8	23.2	↓	214.1	2.1	↓
2013–2017	297.7			253.2			209.5		
Men									
1997–2001	297.1	20.2	↓	246.6	19.9	↓	196.4	7.8	↓
2013–2017	237.0			197.0			180.9		
Women									
1997–2001	344.5	6.4	↓	417.3	25.6	↓	232.0	2.3	↑
2013–2017	322.4			310.1			237.4		
18–29									
1997–2001	280.1	18.9	↓	193.0	74.3	↓	175.0	30.5	↓
2013–2017	226.9			49.5			121.6		
30–39									
1997–2001	222.6	10.1	↓	164.6	60.5	↓	146.9	17.2	↓
2013–2017	200.0			65.0			121.6		
40–49									
1997–2001	226.9	4.71	↓	211.1	43.4	↓	150.6	10.7	↓
2013–2017	216.2			119.3			134.4		
50–59									
1997–2001	300.8	0.7	↑	234.9	21.3	↓	178.8	5.9	↑
2013–2017	303.2			184.7			189.5		
60–69									
1997–2001	379.0	7.8	↓	268.6	18.9	↓	212.8	10.0	↑
2013–2017	349.2			217.8			234.2		
70–79									
1997–2001	473.6	2.8	↓	400.3	26.4	↓	357.6	5.0	↓
2013–2017	460.0			294.5			339.6		
>80									
1997–2001	747.8	12.0	↑	655.9	12.3	↓	784.1	0.2	↑
2013–2017	837.7			574.8			785.9		

IR = incidence rate; PYs = person‐years; T1D = type 1 diabetes mellitus; T2D = type 2 diabetes mellitus.

### IRs by age groups

**Fig. 3:**
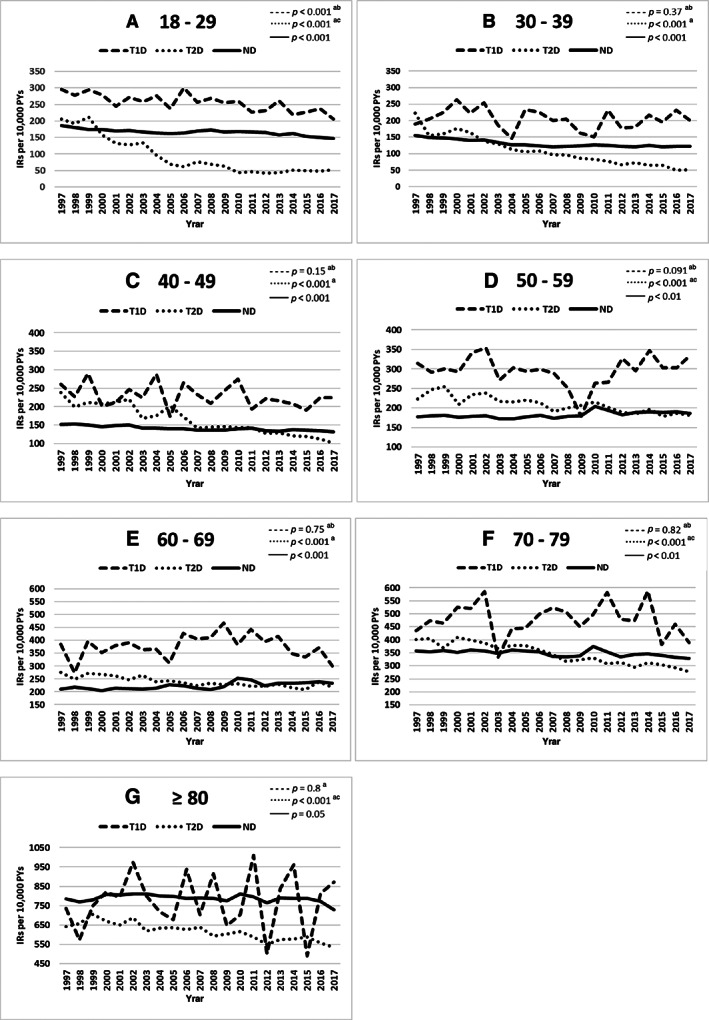
Incidence rates of fractures in Danish adults aged 18 years or older, stratified by diabetes mellitus type and age groups (*A–G*) according to calendar year (1997–2017). The trends in the slope for each group were evaluated using linear regression, and we used pairwise *t* tests to compare the trends among groups. Symbols for the pairwise *t* test: ^a^ *p* < 0.05 for T1D versus T2D, ^b^ *p* < 0.05 for T1D versus ND, and ^c^ *p* < 0.05 for T2D versus ND. IR = incidence rate; PYs = person‐years; ND = without diabetes; T1D = type 1 diabetes mellitus; T2D = type 2 diabetes mellitus.

We observed age‐specific differences in the IRs of fractures in persons with T1D and T2D, and persons without diabetes (Fig. 3*A–G*). The annual counts, IRs, and 95% CIs in different age groups, stratified by diabetes status, are provided in Supplemental Tables S3–S9.^(^ ^25^ ^)^ For persons in the 18‐ to 29‐years age group (Table 2), the median IR of fractures in persons with T1D decreased 18.9%. In the same age group, the decline was most prominent in persons with T2D, where the median IR decreased 74.3% compared with a 30.5% decline in the median IR of fractures in persons without diabetes. A similar decreasing trend was observed for the age groups 30–39 and 40–49 years (Table 2), where the median IRs decreased by 10.1% and 4.71%, respectively, in persons with T1D, and 60.5% and 43.4%, respectively, in persons with T2D, compared with 17.2% and 10.7%, respectively, in persons without diabetes. For persons aged 50–59 years (Table 2), a declining trend of 0.7% was detected for the median IR of fractures in persons with T1D, and the median IR of fractures decreased by 21.3% in persons with T2D but increased 5.9% increase in persons without diabetes. Also, for persons aged 60–69 years (Table 2), the median IR of fractures decreased by 21.3% in persons with T2D and by 7.8% in persons with T1D. By contrast, the median IR in this age group increased by 10% in persons without diabetes. Among persons aged 70 to 79 years (Table 2), the median IR of fractures decreased by 26.4%, 2.8%, and 5% in persons with T2D, T1D, and without diabetes, respectively. However, in the persons aged 80+ years (Table 2), the median IR of fractures increased by 12% in persons with T1D compared with a 12.3% decline in persons with T2D and an increase of 0.2% in persons without diabetes. Linear regression analyses showed declining trends over time in all the groups of persons with T2D (*p*‐trend <0.001) and in persons without diabetes (*p*‐trend <0.01) (Fig. 3*A–G*), but the trends for persons with T1D were not statistically significant except for persons aged 18–29 years (*p*‐trend <0.001) (Fig. 3*A–G*). The trends for persons with T1D and persons with T2D were statistically significantly different in all age groups (*p* < 0.05), with higher declining trends in persons with T1D than in persons with T2D (Fig. 3*A–G*).

### Trends of glucose‐lowering medication use

**Fig. 4:**
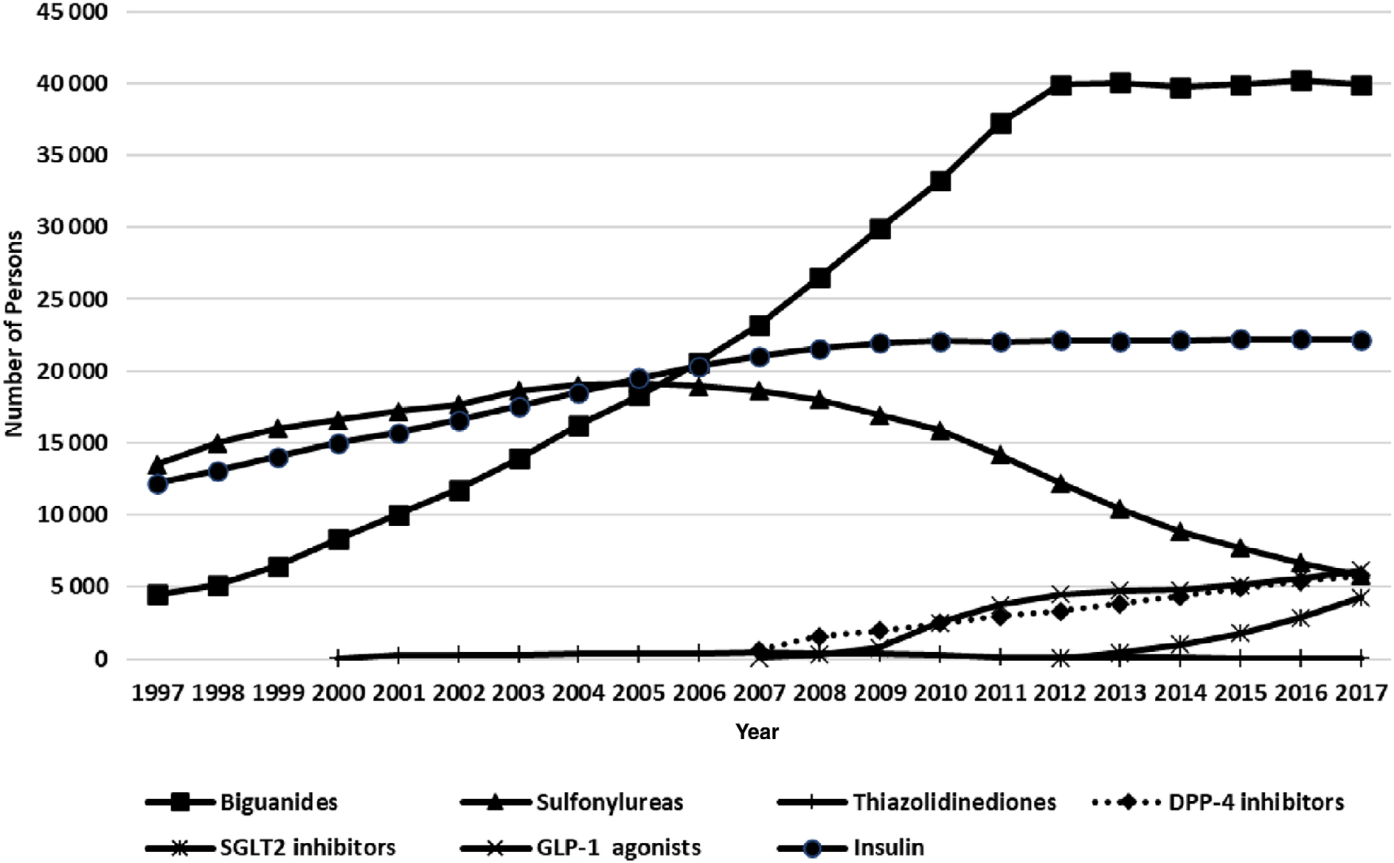
Trends in glucose‐lowering medications use in Danish persons with type 1 and 2 diabetes mellitus according to calendar year (1997–2017). DPP‐4 inhibitors = dipeptidyl peptidase‐4 inhibitors; GLP‐1 = glucagon‐like peptide‐1 agonists; SGLT2 = sodium‐glucose cotransporter‐2 inhibitors.

We noted an increasing utilization of GLP‐1 agonists, SGLT2 inhibitors, and DPP‐4 inhibitors, particularly post‐2007, along with a simultaneous decline in sulfonylureas, thiazolidinediones, and a stable trend of insulin use (Fig. 4).

### Sex‐ and age‐adjusted trends in IRs of fractures

The sensitivity analysis results revealed that the declining IRs of fractures were significant among persons with T2D and persons without diabetes (*p* < 0.01), while we observed a stable trend in IRs of fractures in persons with T1D (*p* = 0.85) (Supplemental Fig. S1).

## Discussion

This study describes the sex‐ and age‐specific trends of fractures in persons with T1D, T2D, and without diabetes between 1997 and 2017. We observed a decreasing trend in the IRs of fractures in men with T1D, T2D, and without diabetes, whereas the trends in the IRs for fractures decreased in women with T2D but not in women with T1D. The trends in the IRs of fractures remained higher and unchanged in persons with T1D in all age groups except for the youngest age group (18–29 years). By contrast, IRs of fractures in persons with T2D decreased to be lower than in those without diabetes in most of the age groups, particularly in persons <50 years and >70 years.

In their study, Abtahi and colleagues^(^ ^20^ ^)^ demonstrated a general decline in osteoporotic fractures such as hip and humerus among adults aged 50+ years in Denmark, from 1995 to 2010, in both men and women. Interestingly, we observed declining trends of fractures in men with T1D, and in men and women with T2D, despite more aged individuals in Denmark,^(^ ^26^ ^)^ suggesting an enhanced diabetes management and increased awareness of bone complications in T1D and T2D.

Despite an increase in the use of anti‐osteoporosis medications in Denmark between 1997 and 2006, the decline in the incidences of hip fracture appears too large to be attributable to the use of anti‐osteoporosis medications alone.^(^ ^27^ ^)^ The decline in the incidence of fractures in persons with T1D and T2D may be partly explained by improvements in diabetes management, increased physical activity and smoking reduction in persons with T1D and T2D,^(^ ^28^ ^)^ and the promotion of use of vitamin D and calcium supplements in persons with T1D and T2D.^(^ ^13^ ^)^ In a repeated cross‐sectional survey,^(^ ^28^ ^)^ physical activity increased in Danish adults with T1D and T2D, particularly in women between 2000 and 2010, which support the reduction in fracture incidences through prevention of sarcopenia and frailty.

Furthermore, we observed a decline in the trends of IRs of fractures after adjustment for age and sex, suggesting that the decline may be explained by improved diabetes management. The introduction of prefilled insulin pens and insulin pumps,^(^ ^29^ ^)^ the improvements in continuous glucose monitoring, and the development of automated insulin‐delivery systems^(^ ^30^ ^)^ may have improved glucose control in T1D, which could contribute to the observed reduction in fracture rates. Also, novel classes of glucose‐lowering medications in T2D might have reduced fracture risk through better glycemic control. Like the trends of glucose‐lowering medications shown in our study, the use of thiazolidinediones and sulfonylureas, associated with an increased fracture risk,^(^ ^31^, ^32^ ^)^ has decreased substantially in persons with T2D in Denmark.^(^ ^33^, ^34^ ^)^ Yet, the use of newer medications such as GLP‐1 agonists and SGLT2 inhibitors, which have neutral or even beneficial effects on fracture risk,^(^ ^35^, ^36^ ^)^ increased rapidly after their introduction.^(^ ^33^, ^34^ ^)^

We observed sex‐ and diabetes‐specific differences in fracture rates. Although rates decreased in men and women with T2D, this was only observed in men with T1D as the rates were steady among women with T1D. Several factors could contribute to the stable trends observed in women with T1D, including improved life expectancy in T1D,^(^ ^37^ ^)^ which could see more women sustaining age‐related bone loss and subsequently increased risk of fragility fractures despite improvements in management. Furthermore, earlier debut of menopause in T1D but reduced usage of hormone‐replacement therapy in Danish women since the early 2000s^(^ ^38^ ^)^ may have affected trends in fracture risk in women with T1D in particular.

Although the trends in IRs of fractures appear to be different among men and women with T1D and T2D, distinct trends were observed among different age groups. A large decline in the IRs of fractures was observed in younger age groups (<50 years old), especially among persons with T2D, possibly because of better glycemic control, earlier detection and screening and in turn better fracture preventive measurements, and fewer traumatic fractures such as road traffic accidents in recent years.^(^ ^39^ ^)^ The decline in the IRs of fractures continued in the older age groups in persons with T2D and to a lesser extent in persons with T1D, which could be attributable to the longer duration of diabetes in persons with T1D and higher prevalence of microvascular complications in T1D such as diabetic retinopathy,^(^ ^40^ ^)^ contributing to higher fracture risk.^(^ ^41^ ^)^ Also, earlier diagnosis of T2D through improved glucose monitoring could reduce fracture risk through enhanced T2D management. In addition, higher life and health expectancy, better access to public transportation and accessibility to buildings, more shifting toward technology‐based roles, better aids to support mobility, and reduced functional limitations in elderly may reduce fracture risk.^(^ ^42^, ^43^ ^)^ Among the oldest men and women with fractures, the unchanged trend in fracture IRs in persons with T1D but declining IRs in persons with T2D or without diabetes may be explained in part by multiple factors. The prevalence of hypoglycemia unawareness, an autonomic failure to detect low blood glucose, is more common in T1D than in T2D,^(^ ^44^ ^)^ which could have an impact on fracture IRs. The stable trend in fracture IRs could at least in theory be related to more cases of hypoglycemia in this age group than in younger age groups with T1D.^(^ ^45^ ^)^

Our study has a number of strengths and limitations. It is based on a longitudinal database that captures all persons residing in Denmark, allowing us to investigate incident fractures over a long observational period. In addition, we used a washout period to minimize overestimation of fractures by counting the same fracture twice. Although the DNPR has a high validity and completeness, the registry data consist of only hospital records including all inpatients and outpatient contacts. Thus, the analyses were based on fractures that only resulted in a hospital contact. Also, low‐energy fractures of the spine may not be identified,^(^ ^46^ ^)^ suggesting the underestimation of vertebral fractures. The classification of T1D and T2D was based on diagnostic and prescription records, which carry the risk of misclassification. For example, some individuals without a diabetes diagnosis code who filled a single non‐insulin glucose‐lowering medication prescription before a fracture may have been misidentified as persons without diabetes, if the T2D code was not registered within 3 months of the fracture. A misclassification is unlikely to influence the observed trends in fractures, considering the large number of persons without diabetes (*n* = 1,165,399). Additionally, other forms of diabetes including latent autoimmune diabetes of adults may be misclassified as T2D in our study. Also, subgrouping persons with T1D in different age categories may question the clinical validity of our results in T1D. However, the generalizability of our findings is strengthened by utilizing a nationwide registry capturing a relatively large number of fractures compared with existing literature. Besides, certain patients with fractures were categorized using the M80 code, which can represent either prevalent or incident fractures, suggesting the possibility of fracture occurrence before developing diabetes. Yet, this discrepancy between prevalent and incident fractures could be pertinent in cases involving T2D, where the diagnosis typically occurs later in life compared with T1D. In addition, body mass index (BMI), biochemical tests such as blood glucose measurements, and lifestyle factors such as physical activity and smoking were not collected in the database, restraining us from describing further differences across the study groups. Moreover, despite the availability of obesity codes in the registry (ICD‐8: 277.99; ICD‐10: E66), these codes are not utilized consistently by clinicians.

Our study showed that despite the decline in the trends of fractures in men with T1D and T2D and in women with T2D, the trend in fracture IRs in women with T1D remains unchanged. The decline in fracture IRs was most prominent in younger age groups (<50 years old). The declining trends may be attributable to improved diabetes management, increased awareness of bone complications in T1D and T2D, increased BMI, and enhanced day‐to‐day activity. The stable trend in fracture incidences in women with T1D requires further attention and investigation into the factors contributing to the higher fracture risk in this group.

## Author Contributions

**Mohamad I. Nasser:** Conceptualization; investigation; methodology; validation; visualization; writing – original draft; writing – review and editing. **Morten Frost:** Conceptualization; investigation; methodology; supervision; validation; writing – review and editing. **Peter Vestergaard:** Conceptualization; investigation; methodology; supervision; validation; writing – review and editing. **Annika Vestergaard Kvist:** Conceptualization; data curation; investigation; methodology; validation; visualization; writing – original draft; writing – review and editing. **Andrea M. Burden:** Conceptualization; investigation; methodology; supervision; validation; writing – review and editing. **Richard Eastell:** Conceptualization; investigation; methodology; supervision; validation; writing – review and editing.

## Disclosures

PV reports consulting fees from Novo Nordisk AS. MF reports research grants from Novo Nordisk Foundation; chairmanship of the Expert Committee on treatment of rare bone diseases of the Danish Medicines Council; consulting fees from Novo Nordisk AS; and receipt of drug and placebo free of charge from Novo Nordisk AS for an investigator‐initiated trial. The professorship of AB was partially endowed by the ETH Foundation and PharmaSuisse; however, there was no connection to the current study. RE receives consultancy funding from Immunodiagnostic Systems, Sandoz, Samsung, Haoma Medica, CL Bio, Biocon, Takeda, meeting presentations for Pharmacosmos, Alexion, and Amgen, and grant funding from Roche, Pharmacosmos, and Alexion. MN and AK have no potential conflicts of interest to declare.

### Peer Review

The peer review history for this article is available at https://www.webofscience.com/api/gateway/wos/peer-review/10.1002/jbm4.10836.

## Supporting information


**Figure S1.** Sex‐ and age‐adjusted trends in incidence rates of fractures in persons with type 1 diabetes mellitus (T1D), type 2 diabetes mellitus (T2D) and without diabetes (ND).
**Table S1.** Incidence rates (IR) of fractures at 10,000 person years (PY) among men with type 1 diabetes mellitus, type 2 diabetes mellitus, and without diabetes, with 95% confidence intervals (CI).
**Table S2.** Incidence rates (IR) of fractures at 10,000 person years (PY) among women with type 1 diabetes mellitus, type 2 diabetes mellitus, and without diabetes, with 95% confidence intervals (CI).
**Table S3.** Incidence rates (IR) of fractures at 10,000 person years (PY) among persons with type 1 diabetes mellitus, type 2 diabetes mellitus, and without diabetes, aged 18–29 years, with 95% confidence intervals (CI).
**Table S4.** Incidence rates (IR) of fractures at 10,000 person years (PY) among patients with type 1 diabetes mellitus, type 2 diabetes mellitus, and without diabetes, aged 30–39 years, with 95% confidence intervals (CI).
**Table S5.** Incidence rates (IR) of fractures at 10,000 person years (PY) among patients with type 1 diabetes mellitus, type 2 diabetes mellitus, and without diabetes, aged 40–49 years, with 95% confidence intervals (CI).
**Table S6.** Incidence rates (IR) of fractures at 10,000 person years (PY) among patients with type 1 diabetes mellitus, type 2 diabetes mellitus, and without diabetes, aged 50–59 years, with 95% confidence intervals (CI).
**Table S7.** Incidence rates (IR) of fractures at 10,000 person years (PY) among patients with type 1 diabetes mellitus, type 2 diabetes mellitus, and without diabetes, aged 60–69 years, with 95% confidence intervals (CI).
**Table S8.** Incidence rates (IR) of fractures at 10,000 person years (PY) among patients with type 1 diabetes mellitus, type 2 diabetes mellitus, and without diabetes, aged 70–79 years, with 95% confidence intervals (CI).
**Table S9.** Incidence rates (IR) of fractures at 10,000 person years (PY) among patients with type 1 diabetes mellitus, type 2 diabetes mellitus, and without diabetes, aged 80+ years, with 95% confidence intervals (CI).

## Data Availability

Restrictions apply to the availability of all data generated or analyzed during this study to preserve patient confidentiality or because they were used under license. The corresponding author will on request detail the restrictions and any conditions applied.
