# Supplementary material for: Sex‐ and Age Group‐Specific Fracture Incidence Rates Trends for Type 1 and 2 Diabetes Mellitus
Source: JBMR Plus. 2023 Oct 27;7(11):e10836. doi: 10.1002/jbm4.10836 (PMC10652176; doi:10.1002/jbm4.10836)
Supplement: Supplementary file 1 — Figure S1. Sex‐ and age‐adjusted trends in incidence rates of fractures in persons with type 1 diabetes mellitus (T1D), type 2 diabetes mellitus (T2D) and without diabetes (ND). Table S1. Incidence rates (IR) of fractures at 10,000 person years (PY) among men with type 1 diabetes mellitus, type 2 diabetes mellitus, and without diabetes, with 95% confidence intervals (CI). Table S2. Incidence rates (IR) of fractures at 10,000 person years (PY) among women with type 1 diabetes mellitus, type 2 diabetes mellitus, and without diabetes, with 95% confidence intervals (CI). Table S3. Incidence rates (IR) of fractures at 10,000 person years (PY) among persons with type 1 diabetes mellitus, type 2 diabetes mellitus, and without diabetes, aged 18–29 years, with 95% confidence intervals (CI). Table S4. Incidence rates (IR) of fractures at 10,000 person years (PY) among patients with type 1 diabetes mellitus, type 2 diabetes mellitus, and without diabetes, aged 30–39 years, with 95% confidence intervals (CI). Table S5. Incidence rates (IR) of fractures at 10,000 person years (PY) among patients with type 1 diabetes mellitus, type 2 diabetes mellitus, and without diabetes, aged 40–49 years, with 95% confidence intervals (CI). Table S6. Incidence rates (IR) of fractures at 10,000 person years (PY) among patients with type 1 diabetes mellitus, type 2 diabetes mellitus, and without diabetes, aged 50–59 years, with 95% confidence intervals (CI). Table S7. Incidence rates (IR) of fractures at 10,000 person years (PY) among patients with type 1 diabetes mellitus, type 2 diabetes mellitus, and without diabetes, aged 60–69 years, with 95% confidence intervals (CI). Table S8. Incidence rates (IR) of fractures at 10,000 person years (PY) among patients with type 1 diabetes mellitus, type 2 diabetes mellitus, and without diabetes, aged 70–79 years, with 95% confidence intervals (CI). Table S9. Incidence rates (IR) of fractures at 10,000 person years (PY) among patients with type 1 diabetes [file JBM4-7-e10836-s001.docx]

**Supplementary tables and figures**

**Figure S1. Sex- and age-adjusted trends in incidence rates of fractures in persons with type 1 diabetes mellitus (T1D), type 2 diabetes mellitus (T2D) and without diabetes (ND)**

**
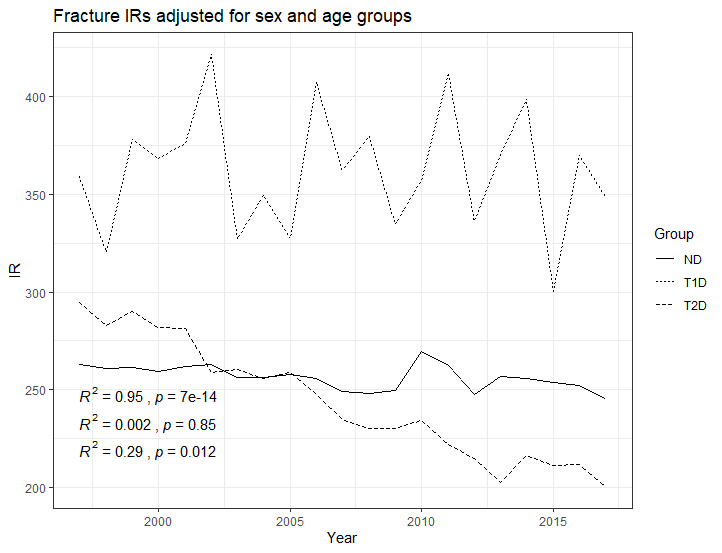
**

**Table S1. Incidence rates (IR) of fractures at 10,000 person years (PY) among men with type 1 diabetes mellitus, type 2 diabetes mellitus, and without diabetes, with 95% confidence intervals (CI).**

|  | **Men** | | | | | | | | |
| --- | --- | --- | --- | --- | --- | --- | --- | --- | --- |
|  | **Type 1 diabetes** | | | **Type 2 diabetes** | | | **Without diabetes** | | |
|  | **N** | **PY at risk** | **IR at 10,000 PY (CI)** | **N** | **PY at risk** | **IR at 10,000 PY (CI)** | **N** | **PY at risk** | **IR at 10,000 PY (CI)** |
| **1997** | 154 | 5,265 | 292.5 (292.0 - 293.0) | 1,308 | 52,278 | 250.2 (250.1 - 250.3) | 39,735 | 1,976,852 | 201.0 (201.0 – 201.0) |
| **1998** | 165 | 5,556 | 297.0 (296.5 - 297.5) | 1,386 | 56,194 | 246.6 (246.5 - 246.7) | 39,187 | 1,978,441 | 198.1 (198.1 - 198.1) |
| **1999** | 184 | 5,787 | 318.0 (317.5 - 318.5) | 1,518 | 60,416 | 251.3 (251.2 - 251.4) | 38,643 | 1,978,402 | 195.3 (195.3 - 195.3) |
| **2000** | 179 | 6,025 | 297.1 (296.7 - 297.5) | 1,591 | 64,578 | 246.4 (246.3 - 246.5) | 38,808 | 1,975,950 | 196.4 (196.4 - 196.4) |
| **2001** | 188 | 6,251 | 300.8 (300.4 - 301.2) | 1,660 | 69,201 | 239.9 (239.8 – 240.0) | 37,914 | 1,973,572 | 192.1 (192.1 - 192.1) |
| **2002** | 222 | 6,525 | 340.2 (339.8 - 340.6) | 1,736 | 73,882 | 235.0 (234.9 - 235.1) | 38,641 | 1,970,250 | 196.1 (196.1 - 196.1) |
| **2003** | 188 | 6,718 | 279.8 (279.4 - 280.2) | 1,793 | 79,543 | 225.4 (225.3 - 225.5) | 37,043 | 1,966,180 | 188.4 (188.4 - 188.4) |
| **2004** | 208 | 6,933 | 300.0 (299.6 - 300.4) | 1,905 | 85,153 | 223.7 (223.6 - 223.8) | 36,569 | 1,963,038 | 186.3 (186.3 - 186.3) |
| **2005** | 191 | 7,163 | 266.6 (266.2 - 267.0) | 2,078 | 90,556 | 229.5 (229.4 - 229.6) | 36,173 | 1,961,443 | 184.4 (184.4 - 184.4) |
| **2006** | 259 | 7,399 | 350.0 (349.6 - 350.4) | 2,070 | 96,427 | 214.7 (214.6 - 214.8) | 35,943 | 1,961,632 | 183.2 (183.2 - 183.2) |
| **2007** | 225 | 7,666 | 293.5 (293.1 - 293.9) | 2,074 | 102,543 | 202.3 (202.2 - 202.4) | 35,983 | 1,964,354 | 183.2 (183.2 - 183.2) |
| **2008** | 217 | 7,908 | 274.4 (274.0 - 274.8) | 2,227 | 109,500 | 203.4 (203.3 - 203.5) | 36,157 | 1,972,428 | 183.3 (183.3 - 183.3) |
| **2009** | 222 | 8,183 | 271.3 (270.9 - 271.7) | 2,506 | 116,979 | 214.2 (214.1 - 214.3) | 35,931 | 1,983,161 | 181.2 (181.2 - 181.2) |
| **2010** | 241 | 8,411 | 286.5 (286.1 - 286.9) | 2,559 | 124,885 | 204.9 (204.8 – 205.0) | 37,176 | 1,987,138 | 187.1 (187.1 - 187.1) |
| **2011** | 262 | 8,643 | 303.1 (302.7 - 303.5) | 2,781 | 134,439 | 206.9 (206.8 – 207.0) | 37,275 | 1,993,077 | 187.0 (187.0 - 187.0) |
| **2012** | 230 | 8,874 | 259.2 (258.9 - 259.5) | 2,732 | 142,179 | 192.2 (192.1 - 192.3) | 36,419 | 1,999,896 | 182.1 (182.1 - 182.1) |
| **2013** | 156 | 9,153 | 170.4 (170.1 - 170.7) | 2,888 | 146,546 | 197.1 (197.0 - 197.2) | 36,295 | 2,013,169 | 180.3 (180.3 - 180.3) |
| **2014** | 271 | 9,394 | 288.5 (288.2 - 288.8) | 3,043 | 150,338 | 202.4 (202.3 - 202.5) | 37,784 | 2,029,263 | 186.2 (186.2 - 186.2) |
| **2015** | 148 | 9,622 | 153.8 (153.6 - 154.0) | 3,020 | 154,819 | 195.1 (195.0 - 195.2) | 37,046 | 2,047,138 | 181.0 (181.0 - 181.0) |
| **2016** | 281 | 9,902 | 283.8 (283.5 - 284.1) | 3,189 | 160,102 | 199.2 (199.1 - 199.3) | 37,532 | 2,069,522 | 181.4 (181.4 - 181.4) |
| **2017** | 241 | 10,166 | 237.1 (236.8 - 237.4) | 3,209 | 164,385 | 195.2 (195.1 - 195.3) | 37,250 | 2,086,709 | 178.5 (178.5 - 178.5) |

**Table S2. Incidence rates (IR) of fractures at 10,000 person years (PY) among women with type 1 diabetes mellitus, type 2 diabetes mellitus, and without diabetes, with 95% confidence intervals (CI).**

|  | **Women** | | | | | | | | |
| --- | --- | --- | --- | --- | --- | --- | --- | --- | --- |
|  | **Type 1 diabetes** | | | **Type 2 diabetes** | | | **Without diabetes** | | |
|  | **N** | **PY at risk** | **IR at 10,000 PY (CI)** | **N** | **PY at risk** | **IR at 10,000 PY (CI)** | **N** | **PY at risk** | **IR at 10,000 PY (CI)** |
| **1997** | 141 | 3,921 | 359.6 (359.0 - 360.2) | 2,164 | 50,104 | 431.9 (431.7 - 432.1) | 48,458 | 2,072,908 | 233.8 (233.8 - 233.8) |
| **1998** | 114 | 4,086 | 279.0 (278.5 - 279.5) | 2,231 | 53,461 | 417.3 (417.1 - 417.5) | 48,126 | 2,073,964 | 232.0 (232.0 - 232.0) |
| **1999** | 146 | 4,237 | 344.6 (344.0 - 345.2) | 2,430 | 57,084 | 425.7 (425.5 - 425.9) | 48,150 | 2,074,675 | 232.1 (232.1 - 232.1) |
| **2000** | 153 | 4,438 | 344.7 (344.2 - 345.2) | 2,525 | 60,800 | 415.3 (415.1 - 415.5) | 46,965 | 2,072,314 | 226.6 (226.6 - 226.6) |
| **2001** | 150 | 4,597 | 326.3 (325.8 - 326.8) | 2,690 | 64,606 | 416.4 (416.2 - 416.6) | 48,109 | 2,069,964 | 232.4 (232.4 - 232.4) |
| **2002** | 166 | 4,721 | 351.6 (351.1 - 352.1) | 2,870 | 68,568 | 418.6 (418.4 - 418.8) | 47,753 | 2,067,482 | 231.0 (231.0 - 231.0) |
| **2003** | 135 | 4,855 | 278.1 (277.6 - 278.6) | 2,827 | 73,701 | 383.6 (383.5 - 383.7) | 46,575 | 2,063,207 | 225.7 (225.7 - 225.7) |
| **2004** | 149 | 5,008 | 297.5 (297.0 - 298.0) | 2,987 | 79,222 | 377.0 (376.9 - 377.1) | 46,635 | 2,059,324 | 226.5 (226.5 - 226.5) |
| **2005** | 152 | 5,200 | 292.3 (291.8 - 292.8) | 3,197 | 84,962 | 376.3 (376.2 - 376.4) | 47,678 | 2,056,592 | 231.8 (231.8 - 231.8) |
| **2006** | 169 | 5,363 | 315.1 (314.6 - 315.6) | 3,325 | 90,860 | 365.9 (365.8 - 366.0) | 47,620 | 2,055,212 | 231.7 (231.7 - 231.7) |
| **2007** | 175 | 5,556 | 315.0 (314.5 - 315.5) | 3,420 | 97,454 | 350.9 (350.8 - 351.0) | 46,264 | 2,055,601 | 225.1 (225.1 - 225.1) |
| **2008** | 196 | 5,747 | 341.0 (340.5 - 341.5) | 3,484 | 104,406 | 333.7 (333.6 - 333.8) | 46,652 | 2,060,318 | 226.4 (226.4 - 226.4) |
| **2009** | 171 | 5,946 | 287.6 (287.2 - 288.0) | 3,614 | 111,793 | 323.3 (323.2 - 323.4) | 47,663 | 2,068,184 | 230.5 (230.5 - 230.5) |
| **2010** | 184 | 6,173 | 298.1 (297.7 - 298.5) | 4,078 | 119,351 | 341.7 (341.6 - 341.8) | 52,794 | 2,073,270 | 254.6 (254.6 - 254.6) |
| **2011** | 202 | 6,379 | 316.7 (316.3 - 317.1) | 4,000 | 128,424 | 311.5 (311.4 - 311.6) | 50,841 | 2,078,634 | 244.6 (244.6 - 244.6) |
| **2012** | 207 | 6,587 | 314.3 (313.9 - 314.7) | 4,252 | 136,234 | 312.1 (312.0 - 312.2) | 47,758 | 2,084,457 | 229.1 (229.1 - 229.1) |
| **2013** | 221 | 6,854 | 322.4 (322.0 - 322.8) | 4,409 | 141,549 | 311.5 (311.4 - 311.6) | 49,738 | 2,095,056 | 237.4 (237.4 - 237.4) |
| **2014** | 234 | 7,073 | 330.8 (330.4 - 331.2) | 4,553 | 145,993 | 311.9 (311.8 - 312.0) | 50,086 | 2,107,750 | 237.6 (237.6 - 237.6) |
| **2015** | 194 | 7,320 | 265.0 (264.6 - 265.4) | 4,699 | 151,498 | 310.2 (310.1 - 310.3) | 50,520 | 2,119,424 | 238.4 (238.4 - 238.4) |
| **2016** | 241 | 7,630 | 315.9 (315.5 - 316.3) | 4,851 | 157,220 | 308.5 (308.4 - 308.6) | 50,584 | 2,135,415 | 236.9 (236.9 - 236.9) |
| **2017** | 265 | 7,937 | 333.9 (333.5 - 334.3) | 4,661 | 162,665 | 286.5 (286.4 - 286.6) | 49,966 | 2,148,685 | 232.5 (232.5 - 232.5) |

**Table S3. Incidence rates (IR) of fractures at 10,000 person years (PY) among persons with type 1 diabetes mellitus, type 2 diabetes mellitus, and without diabetes, aged 18-29 years, with 95% confidence intervals (CI).**

|  | **18 - 29** | | | | | | | | |
| --- | --- | --- | --- | --- | --- | --- | --- | --- | --- |
|  | **Type 1 diabetes** | | | **Type 2 diabetes** | | | **Without diabetes** | | |
|  | **N** | **PY at risk** | **IR at 10,000 PY (CI)** | **N** | **PY at risk** | **IR at 10,000 PY (CI)** | **N** | **PY at risk** | **IR at 10,000 PY (CI)** |
| **1997** | 51 | 1,727 | 295.3 (294.5 - 296.1) | 72 | 3,509 | 205.2 (204.7 - 205.7) | 16,475 | 881,634 | 186.9 (186.9 - 186.9) |
| **1998** | 51 | 1,833 | 278.2 (277.4 - 279.0) | 69 | 3,574 | 193.1 (192.6 - 193.6) | 15,617 | 863,397 | 180.9 (180.9 - 180.9) |
| **1999** | 56 | 1,898 | 295.0 (294.2 - 295.8) | 79 | 3,717 | 212.5 (212.0 - 213.0) | 14,822 | 849,332 | 174.5 (174.5 - 174.5) |
| **2000** | 56 | 1,999 | 280.1 (279.4 - 280.8) | 61 | 3,858 | 158.1 (157.7 - 158.5) | 14,593 | 833,897 | 175.0 (175.0 - 175.0) |
| **2001** | 51 | 2,080 | 245.2 (244.5 - 245.9) | 51 | 3,878 | 131.5 (131.1 - 131.9) | 13,915 | 817,837 | 170.1 (170.1 - 170.1) |
| **2002** | 59 | 2,167 | 272.3 (271.6 - 273.0) | 52 | 4,071 | 127.7 (127.4 - 128.0) | 13,692 | 797,794 | 171.6 (171.6 - 171.6) |
| **2003** | 58 | 2,250 | 257.8 (257.1 - 258.5) | 58 | 4,340 | 133.6 (133.3 - 133.9) | 12,983 | 777,604 | 167.0 (167.0 - 167.0) |
| **2004** | 64 | 2,320 | 275.9 (275.2 - 276.6) | 45 | 4,696 | 95.8 (95.5 - 96.1) | 12,460 | 762,122 | 163.5 (163.5 - 163.5) |
| **2005** | 57 | 2,394 | 238.1 (237.5 - 238.7) | 36 | 5,222 | 68.9 (68.7 - 69.1) | 12,119 | 747,878 | 162.0 (162.0 - 162.0) |
| **2006** | 75 | 2,498 | 300.2 (299.5 - 300.9) | 36 | 5,817 | 61.9 (61.7 - 62.1) | 12,016 | 736,000 | 163.3 (163.3 - 163.3) |
| **2007** | 66 | 2,576 | 256.2 (255.6 - 256.8) | 50 | 6,462 | 77.4 (77.2 - 77.6) | 12,446 | 734,103 | 169.5 (169.5 - 169.5) |
| **2008** | 71 | 2,647 | 268.2 (267.6 - 268.8) | 49 | 7,066 | 69.3 (69.1 - 69.5) | 12,792 | 742,503 | 172.3 (172.3 - 172.3) |
| **2009** | 70 | 2,734 | 256.0 (255.4 - 256.6) | 49 | 7,738 | 63.3 (63.1 - 63.5) | 12,603 | 754,154 | 167.1 (167.1 - 167.1) |
| **2010** | 75 | 2,879 | 260.5 (259.9 - 261.1) | 36 | 8,190 | 44.0 (43.9 - 44.1) | 12,855 | 763,172 | 168.4 (168.4 - 168.4) |
| **2011** | 69 | 3,034 | 227.4 (226.9 - 227.9) | 40 | 8,622 | 46.4 (46.3 - 46.5) | 13,005 | 777,872 | 167.2 (167.2 - 167.2) |
| **2012** | 75 | 3,232 | 232.1 (231.6 - 232.6) | 38 | 9,029 | 42.1 (42.0 - 42.2) | 13,142 | 794,698 | 165.4 (165.4 - 165.4) |
| **2013** | 90 | 3,443 | 261.4 (260.9 - 261.9) | 41 | 9,304 | 44.1 (44.0 - 44.2) | 12,878 | 814,619 | 158.1 (158.1 - 158.1) |
| **2014** | 79 | 3,612 | 218.7 (218.2 - 219.2) | 49 | 9,457 | 51.8 (51.7 - 51.9) | 13,521 | 836,904 | 161.6 (161.6 - 161.6) |
| **2015** | 87 | 3,833 | 227.0 (226.5 - 227.5) | 48 | 9,697 | 49.5 (49.4 - 49.6) | 13,064 | 855,525 | 152.7 (152.7 - 152.7) |
| **2016** | 96 | 4,053 | 236.9 (236.4 - 237.4) | 48 | 10,025 | 47.9 (47.8 - 48.0) | 13,139 | 878,432 | 149.6 (149.6 - 149.6) |
| **2017** | 86 | 4,180 | 205.7 (205.3 - 206.1) | 55 | 10,343 | 53.2 (53.1 - 53.3) | 13,149 | 893,612 | 147.1 (147.1 - 147.1) |

**Table S4. Incidence rates (IR) of fractures at 10,000 person years (PY) among patients with type 1 diabetes mellitus, type 2 diabetes mellitus, and without diabetes, aged 30-39 years, with 95% confidence intervals (CI).**

|  | **30 - 39** | | | | | | | | | |
| --- | --- | --- | --- | --- | --- | --- | --- | --- | --- | --- |
|  | **Type 1 diabetes** | | | **Type 2 diabetes** | | | **Without diabetes** | | | |
|  | **N** | **PY at risk** | **IR at 10,000 PY (CI)** | **N** | **PY at risk** | **IR at 10,000 PY (CI)** | **N** | **PY at risk** | **IR at 10,000 PY (CI)** | |
| **1997** | 33 | 1,746 | 189.0 (188.4 - 189.6) | 157 | 7,056 | 222.5 (222.2 - 222.8) | 12,359 | 794,800 | | 155.5 (155.5 - 155.5) |
| **1998** | 38 | 1,862 | 204.1 (203.5 - 204.7) | 119 | 7,653 | 155.5 (155.2 - 155.8) | 11,978 | 803,934 | | 149.0 (149.0 - 149.0) |
| **1999** | 45 | 2,008 | 224.1 (223.4 - 224.8) | 131 | 8,171 | 160.3 (160.0 - 160.6) | 11,853 | 806,547 | | 147.0 (147.0 - 147.0) |
| **2000** | 55 | 2,090 | 263.2 (262.5 - 263.9) | 152 | 8,628 | 176.2 (175.9 - 176.5) | 11,651 | 806,726 | | 144.4 (144.4 - 144.4) |
| **2001** | 49 | 2,201 | 222.6 (222.0 - 223.2) | 149 | 9,050 | 164.6 (164.3 - 164.9) | 11,230 | 804,269 | | 139.6 (139.6 - 139.6) |
| **2002** | 58 | 2,287 | 253.6 (252.9 - 254.3) | 130 | 9,426 | 137.9 (137.7 - 138.1) | 11,403 | 805,141 | | 141.6 (141.6 - 141.6) |
| **2003** | 44 | 2,348 | 187.4 (186.8 - 188.0) | 130 | 10,061 | 129.2 (129.0 - 129.4) | 10,738 | 804,444 | | 133.5 (133.5 - 133.5) |
| **2004** | 35 | 2,392 | 146.3 (145.8 - 146.8) | 122 | 10,730 | 113.7 (113.5 - 113.9) | 10,126 | 795,343 | | 127.3 (127.3 - 127.3) |
| **2005** | 58 | 2,483 | 233.6 (233.0 - 234.2) | 121 | 11,486 | 105.3 (105.1 - 105.5) | 9,915 | 784,137 | | 126.4 (126.4 - 126.4) |
| **2006** | 58 | 2,565 | 226.1 (225.5 - 226.7) | 133 | 12,221 | 108.8 (108.6 - 109.0) | 9,535 | 771,993 | | 123.5 (123.5 - 123.5) |
| **2007** | 53 | 2,642 | 200.6 (200.1 - 201.1) | 126 | 13,124 | 96.0 (95.8 - 96.2) | 9,067 | 751,828 | | 120.6 (120.6 - 120.6) |
| **2008** | 56 | 2,742 | 204.2 (203.7 - 204.7) | 137 | 14,155 | 96.8 (96.6 - 97.0) | 9,052 | 737,665 | | 122.7 (122.7 - 122.7) |
| **2009** | 46 | 2,828 | 162.7 (162.2 - 163.2) | 133 | 15,419 | 86.3 (86.2 - 86.4) | 9,012 | 730,059 | | 123.4 (123.4 - 123.4) |
| **2010** | 44 | 2,920 | 150.7 (150.3 - 151.1) | 139 | 16,629 | 83.6 (83.5 - 83.7) | 9,165 | 721,213 | | 127.1 (127.1 - 127.1) |
| **2011** | 69 | 2,977 | 231.8 (231.3 - 232.3) | 137 | 17,818 | 76.9 (76.8 - 77.0) | 8,867 | 711,586 | | 124.6 (124.6 - 124.6) |
| **2012** | 53 | 2,992 | 177.1 (176.6 - 177.6) | 124 | 18,702 | 66.3 (66.2 - 66.4) | 8,486 | 694,342 | | 122.2 (122.2 - 122.2) |
| **2013** | 55 | 3,042 | 180.8 (180.3 - 181.3) | 140 | 19,467 | 71.9 (71.8 - 72.0) | 8,161 | 677,525 | | 120.5 (120.5 - 120.5) |
| **2014** | 66 | 3,050 | 216.4 (215.9 - 216.9) | 133 | 20,276 | 65.6 (65.5 - 65.7) | 8,299 | 664,499 | | 124.9 (124.9 - 124.9) |
| **2015** | 61 | 3,108 | 196.3 (195.8 - 196.8) | 137 | 21,056 | 65.1 (65.0 - 65.2) | 7,866 | 654,570 | | 120.2 (120.2 - 120.2) |
| **2016** | 73 | 3,144 | 232.2 (231.7 - 232.7) | 109 | 22,045 | 49.4 (49.3 - 49.5) | 7,886 | 648,404 | | 121.6 (121.6 - 121.6) |
| **2017** | 65 | 3,249 | 200.1 (199.6 - 200.6) | 120 | 23,137 | 51.9 (51.8 - 52.0) | 7,888 | 647,583 | | 121.8 (121.8 - 121.8) |

**Table S5. Incidence rates (IR) of fractures at 10,000 person years (PY) among patients with type 1 diabetes mellitus, type 2 diabetes mellitus, and without diabetes, aged 40-49 years, with 95% confidence intervals (CI).**

|  | **40 - 49** | | | | | | | | | |
| --- | --- | --- | --- | --- | --- | --- | --- | --- | --- | --- |
|  | **Type 1 diabetes** | | | **Type 2 diabetes** | | | **Without diabetes** | | | |
|  | **N** | **PY at risk** | **IR at 10,000 PY (CI)** | **N** | **PY at risk** | **IR at 10,000 PY (CI)** | **N** | **PY at risk** | **IR at 10,000 PY (CI)** | |
| **1997** | 45 | 1,728 | 260.4 (259.6 - 261.2) | 266 | 11,150 | 238.6 (238.3 - 238.9) | 11,243 | 743,032 | | 151.3 (151.3 - 151.3) |
| **1998** | 42 | 1,851 | 226.9 (226.2 - 227.6) | 237 | 11,897 | 199.2 (198.9 - 199.5) | 11,184 | 732,204 | | 152.7 (152.7 - 152.7) |
| **1999** | 56 | 1,928 | 290.5 (289.7 - 291.3) | 268 | 12,693 | 211.1 (210.8 - 211.4) | 10,951 | 726,814 | | 150.7 (150.7 - 150.7) |
| **2000** | 41 | 2,035 | 201.5 (200.9 - 202.1) | 284 | 13,653 | 208.0 (207.8 - 208.2) | 10,626 | 724,771 | | 146.6 (146.6 - 146.6) |
| **2001** | 45 | 2,141 | 210.2 (209.6 - 210.8) | 311 | 14,713 | 211.4 (211.2 - 211.6) | 10,839 | 725,512 | | 149.4 (149.4 - 149.4) |
| **2002** | 55 | 2,230 | 246.6 (245.9 - 247.3) | 349 | 15,813 | 220.7 (220.5 - 220.9) | 10,918 | 728,803 | | 149.8 (149.8 - 149.8) |
| **2003** | 51 | 2,277 | 224.0 (223.4 - 224.6) | 286 | 16,957 | 168.7 (168.5 - 168.9) | 10,388 | 732,795 | | 141.8 (141.8 - 141.8) |
| **2004** | 68 | 2,361 | 288.0 (287.3 - 288.7) | 319 | 18,196 | 175.3 (175.1 - 175.5) | 10,519 | 739,766 | | 142.2 (142.2 - 142.2) |
| **2005** | 42 | 2,430 | 172.8 (172.3 - 173.3) | 391 | 19,401 | 201.5 (201.3 - 201.7) | 10,564 | 749,798 | | 140.9 (140.9 - 140.9) |
| **2006** | 66 | 2,496 | 264.4 (263.8 - 265.0) | 356 | 20,664 | 172.3 (172.1 - 172.5) | 10,626 | 760,575 | | 139.7 (139.7 - 139.7) |
| **2007** | 61 | 2,627 | 232.2 (231.6 - 232.8) | 312 | 22,029 | 141.6 (141.4 - 141.8) | 10,573 | 773,632 | | 136.7 (136.7 - 136.7) |
| **2008** | 57 | 2,719 | 209.6 (209.1 - 210.1) | 341 | 23,559 | 144.7 (144.5 - 144.9) | 10,642 | 781,926 | | 136.1 (136.1 - 136.1) |
| **2009** | 70 | 2,862 | 244.6 (244.0 - 245.2) | 368 | 25,217 | 145.9 (145.8 - 146.0) | 10,675 | 785,048 | | 136.0 (136.0 - 136.0) |
| **2010** | 80 | 2,910 | 274.9 (274.3 - 275.5) | 381 | 26,735 | 142.5 (142.4 - 142.6) | 11,031 | 784,763 | | 140.6 (140.6 - 140.6) |
| **2011** | 58 | 2,990 | 194.0 (193.5 - 194.5) | 406 | 28,410 | 142.9 (142.8 - 143.0) | 11,127 | 781,810 | | 142.3 (142.3 - 142.3) |
| **2012** | 68 | 3,068 | 221.6 (221.1 - 222.1) | 377 | 29,579 | 127.5 (127.4 - 127.6) | 10,547 | 782,128 | | 134.9 (134.9 - 134.9) |
| **2013** | 68 | 3,145 | 216.2 (215.7 - 216.7) | 391 | 30,377 | 128.7 (128.6 - 128.8) | 10,443 | 782,372 | | 133.5 (133.5 - 133.5) |
| **2014** | 66 | 3,174 | 207.9 (207.4 - 208.4) | 376 | 31,017 | 121.2 (121.1 - 121.3) | 10,627 | 775,564 | | 137.0 (137.0 - 137.0) |
| **2015** | 62 | 3,239 | 191.4 (190.9 - 191.9) | 379 | 31,765 | 119.3 (119.2 - 119.4) | 10,420 | 768,204 | | 135.6 (135.6 - 135.6) |
| **2016** | 75 | 3,335 | 224.9 (224.4 - 225.4) | 365 | 32,622 | 111.9 (111.8 - 112.0) | 10,233 | 761,202 | | 134.4 (134.4 - 134.4) |
| **2017** | 75 | 3,348 | 224.0 (223.5 - 224.5) | 340 | 33,479 | 101.6 (101.5 - 101.7) | 9,839 | 744,885 | | 132.1 (132.1 - 132.1) |

**Table S6. Incidence rates (IR) of fractures at 10,000 person years (PY) among patients with type 1 diabetes mellitus, type 2 diabetes mellitus, and without diabetes, aged 50-59 years, with 95% confidence intervals (CI).**

|  | **50 - 59** | | | | | | | | | |
| --- | --- | --- | --- | --- | --- | --- | --- | --- | --- | --- |
|  | **Type 1 diabetes** | | | **Type 2 diabetes** | | | **Without diabetes** | | | |
|  | **N** | **PY at risk** | **IR at 10,000 PY (CI)** | **N** | **PY at risk** | **IR at 10,000 PY (CI)** | **N** | **PY at risk** | **IR at 10,000 PY (CI)** | |
| **1997** | 46 | 1,461 | 314.9 (314.0 - 315.8) | 426 | 19,107 | 223.0 (222.8 - 223.2) | 11,720 | 657,784 | | 178.2 (178.2 - 178.2) |
| **1998** | 45 | 1,542 | 291.8 (290.9 - 292.7) | 524 | 21,209 | 247.1 (246.9 - 247.3) | 12,262 | 680,907 | | 180.1 (180.1 - 180.1) |
| **1999** | 49 | 1,629 | 300.8 (300.0 - 301.6) | 597 | 23,456 | 254.5 (254.3 - 254.7) | 12,620 | 696,829 | | 181.1 (181.1 - 181.1) |
| **2000** | 51 | 1,741 | 292.9 (292.1 - 293.7) | 534 | 25,518 | 209.3 (209.1 - 209.5) | 12,501 | 708,565 | | 176.4 (176.4 - 176.4) |
| **2001** | 62 | 1,811 | 342.4 (341.5 - 343.3) | 654 | 27,832 | 235.0 (234.8 - 235.2) | 12,841 | 717,977 | | 178.8 (178.8 - 178.8) |
| **2002** | 67 | 1,893 | 353.9 (353.1 - 354.7) | 706 | 29,690 | 237.8 (237.6 - 238.0) | 13,046 | 723,812 | | 180.2 (180.2 - 180.2) |
| **2003** | 53 | 1,955 | 271.1 (270.4 - 271.8) | 690 | 31,770 | 217.2 (217.0 - 217.4) | 12,511 | 723,508 | | 172.9 (172.9 - 172.9) |
| **2004** | 62 | 2,046 | 303.0 (302.2 - 303.8) | 721 | 33,426 | 215.7 (215.5 - 215.9) | 12,480 | 720,931 | | 173.1 (173.1 - 173.1) |
| **2005** | 62 | 2,105 | 294.5 (293.8 - 295.2) | 766 | 34,653 | 221.0 (220.8 - 221.2) | 12,682 | 712,019 | | 178.1 (178.1 - 178.1) |
| **2006** | 64 | 2,138 | 299.3 (298.6 - 300.0) | 762 | 35,751 | 213.1 (212.9 - 213.3) | 12,707 | 700,896 | | 181.3 (181.3 - 181.3) |
| **2007** | 64 | 2,210 | 289.6 (288.9 - 290.3) | 707 | 37,037 | 190.9 (190.8 - 191.0) | 11,995 | 687,992 | | 174.3 (174.3 - 174.3) |
| **2008** | 57 | 2,269 | 251.2 (250.5 - 251.9) | 772 | 38,469 | 200.7 (200.6 - 200.8) | 12,076 | 677,255 | | 178.3 (178.3 - 178.3) |
| **2009** | 42 | 2,350 | 178.7 (178.2 - 179.2) | 835 | 40,467 | 206.3 (206.2 - 206.4) | 12,131 | 671,812 | | 180.6 (180.6 - 180.6) |
| **2010** | 64 | 2,422 | 264.2 (263.6 - 264.8) | 924 | 42,930 | 215.2 (215.1 - 215.3) | 13,691 | 668,949 | | 204.7 (204.7 - 204.7) |
| **2011** | 67 | 2,517 | 266.2 (265.6 - 266.8) | 927 | 46,006 | 201.5 (201.4 - 201.6) | 12,939 | 668,245 | | 193.6 (193.6 - 193.6) |
| **2012** | 84 | 2,566 | 327.4 (326.7 - 328.1) | 917 | 48,558 | 188.8 (188.7 - 188.9) | 12,238 | 670,396 | | 182.5 (182.5 - 182.5) |
| **2013** | 78 | 2,640 | 295.5 (294.8 - 296.2) | 915 | 49,528 | 184.7 (184.6 - 184.8) | 12,730 | 675,583 | | 188.4 (188.4 - 188.4) |
| **2014** | 96 | 2,760 | 347.8 (347.1 - 348.5) | 990 | 50,440 | 196.3 (196.2 - 196.4) | 12,994 | 684,342 | | 189.9 (189.9 - 189.9) |
| **2015** | 86 | 2,836 | 303.2 (302.6 - 303.8) | 932 | 52,105 | 178.9 (178.8 - 179.0) | 13,192 | 695,937 | | 189.6 (189.6 - 189.6) |
| **2016** | 88 | 2,910 | 302.4 (301.8 - 303.0) | 1,005 | 54,027 | 186.0 (185.9 - 186.1) | 13,498 | 708,311 | | 190.6 (190.6 - 190.6) |
| **2017** | 101 | 3,013 | 335.2 (334.5 - 335.9) | 1,011 | 55,682 | 181.6 (181.5 - 181.7) | 13,502 | 723,008 | | 186.7 (186.7 - 186.7) |

**Table S7. Incidence rates (IR) of fractures at 10,000 person years (PY) among patients with type 1 diabetes mellitus, type 2 diabetes mellitus, and without diabetes, aged 60-69 years, with 95% confidence intervals (CI).**

|  | **60 - 69** | | | | | | | | | |
| --- | --- | --- | --- | --- | --- | --- | --- | --- | --- | --- |
|  | **Type 1 diabetes** | | | **Type 2 diabetes** | | | **Without diabetes** | | | |
|  | **N** | **PY at risk** | **IR at 10,000 PY (CI)** | **N** | **PY at risk** | **IR at 10,000 PY (CI)** | **N** | **PY at risk** | **IR at 10,000 PY (CI)** | |
| **1997** | 43 | 1,114 | 386.0 (384.8 - 387.2) | 615 | 22,379 | 274.8 (274.6 - 275.0) | 9,253 | 440,100 | | 210.2 (210.2 - 210.2) |
| **1998** | 31 | 1,135 | 273.1 (272.1 - 274.1) | 604 | 24,296 | 248.6 (248.4 - 248.8) | 9,629 | 442,118 | | 217.8 (217.8 - 217.8) |
| **1999** | 46 | 1,161 | 396.2 (395.1 - 397.3) | 709 | 26,152 | 271.1 (270.9 - 271.3) | 9,467 | 444,886 | | 212.8 (212.8 - 212.8) |
| **2000** | 43 | 1,219 | 352.7 (351.6 - 353.8) | 759 | 28,252 | 268.7 (268.5 - 268.9) | 9,215 | 449,255 | | 205.1 (205.1 - 205.1) |
| **2001** | 47 | 1,240 | 379.0 (377.9 - 380.1) | 800 | 30,408 | 263.1 (262.9 - 263.3) | 9,714 | 455,388 | | 213.3 (213.3 - 213.3) |
| **2002** | 50 | 1,278 | 391.2 (390.1 - 392.3) | 820 | 33,184 | 247.1 (246.9 - 247.3) | 9,872 | 463,078 | | 213.2 (213.2 - 213.2) |
| **2003** | 49 | 1,348 | 363.5 (362.5 - 364.5) | 976 | 36,953 | 264.1 (263.9 - 264.3) | 9,992 | 476,101 | | 209.9 (209.9 - 209.9) |
| **2004** | 51 | 1,394 | 365.9 (364.9 - 366.9) | 980 | 40,828 | 240.0 (239.8 - 240.2) | 10,555 | 493,298 | | 214.0 (214.0 - 214.0) |
| **2005** | 46 | 1,473 | 312.3 (311.4 - 313.2) | 1,086 | 44,769 | 242.6 (242.5 - 242.7) | 11,707 | 514,450 | | 227.6 (227.6 - 227.6) |
| **2006** | 67 | 1,567 | 427.6 (426.6 - 428.6) | 1,159 | 49,276 | 235.2 (235.1 - 235.3) | 12,046 | 537,676 | | 224.0 (224.0 - 224.0) |
| **2007** | 67 | 1,649 | 406.3 (405.3 - 407.3) | 1,209 | 53,834 | 224.6 (224.5 - 224.7) | 11,981 | 561,449 | | 213.4 (213.4 - 213.4) |
| **2008** | 70 | 1,706 | 410.3 (409.3 - 411.3) | 1,366 | 58,557 | 233.3 (233.2 - 233.4) | 12,135 | 580,506 | | 209.0 (209.0 - 209.0) |
| **2009** | 82 | 1,756 | 467.0 (466.0 - 468.0) | 1,438 | 63,145 | 227.7 (227.6 - 227.8) | 13,049 | 593,229 | | 220.0 (220.0 - 220.0) |
| **2010** | 71 | 1,855 | 382.7 (381.8 - 383.6) | 1,567 | 67,720 | 231.4 (231.3 - 231.5) | 15,206 | 601,923 | | 252.6 (252.6 - 252.6) |
| **2011** | 84 | 1,901 | 441.9 (441.0 - 442.8) | 1,625 | 73,330 | 221.6 (221.5 - 221.7) | 15,022 | 607,494 | | 247.3 (247.3 - 247.3) |
| **2012** | 78 | 1,978 | 394.3 (393.4 - 395.2) | 1,711 | 76,908 | 222.5 (222.4 - 222.6) | 13,673 | 611,832 | | 223.5 (223.5 - 223.5) |
| **2013** | 85 | 2,046 | 415.4 (414.5 - 416.3) | 1,797 | 77,991 | 230.4 (230.3 - 230.5) | 14,344 | 613,479 | | 233.8 (233.8 - 233.8) |
| **2014** | 74 | 2,119 | 349.2 (348.4 - 350.0) | 1,694 | 78,217 | 216.6 (216.5 - 216.7) | 14,383 | 613,958 | | 234.3 (234.3 - 234.3) |
| **2015** | 71 | 2,118 | 335.2 (334.4 - 336.0) | 1,629 | 78,197 | 208.3 (208.2 - 208.4) | 14,279 | 608,382 | | 234.7 (234.7 - 234.7) |
| **2016** | 81 | 2,174 | 372.6 (371.8 - 373.4) | 1,863 | 78,026 | 238.8 (238.7 - 238.9) | 14,339 | 601,007 | | 238.6 (238.6 - 238.6) |
| **2017** | 68 | 2,259 | 301.0 (300.3 - 301.7) | 1,692 | 77,664 | 217.9 (217.8 - 218.0) | 13,808 | 592,229 | | 233.2 (233.2 - 233.2) |

**Table S8. Incidence rates (IR) of fractures at 10,000 person years (PY) among patients with type 1 diabetes mellitus, type 2 diabetes mellitus, and without diabetes, aged 70-79 years, with 95% confidence intervals (CI).**

|  | **70 - 79** | | | | | | | | | |
| --- | --- | --- | --- | --- | --- | --- | --- | --- | --- | --- |
|  | **Type 1 diabetes** | | | **Type 2 diabetes** | | | **Without diabetes** | | | |
|  | **N** | **PY at risk** | **IR at 10,000 PY (CI)** | **N** | **PY at risk** | **IR at 10,000 PY (CI)** | **N** | **PY at risk** | **IR at 10,000 PY (CI)** | |
| **1997** | 39 | 894 | 436.2 (434.8 - 437.6) | 958 | 23,928 | 400.4 (400.1 - 400.7) | 12,250 | 342,481 | | 357.7 (357.6 - 357.8) |
| **1998** | 44 | 929 | 473.6 (472.2 - 475.0) | 1,011 | 24,972 | 404.9 (404.7 - 405.1) | 12,036 | 339,961 | | 354.0 (353.9 - 354.1) |
| **1999** | 43 | 928 | 463.4 (462.0 - 464.8) | 976 | 26,445 | 369.1 (368.9 - 369.3) | 12,146 | 337,253 | | 360.1 (360.0 - 360.2) |
| **2000** | 48 | 916 | 524.0 (522.5 - 525.5) | 1,128 | 27,570 | 409.1 (408.9 - 409.3) | 11,801 | 334,575 | | 352.7 (352.6 - 352.8) |
| **2001** | 48 | 920 | 521.7 (520.2 - 523.2) | 1,149 | 28,842 | 398.4 (398.2 - 398.6) | 11,868 | 328,525 | | 361.3 (361.2 - 361.4) |
| **2002** | 55 | 939 | 585.7 (584.2 - 587.2) | 1,172 | 30,231 | 387.7 (387.5 - 387.9) | 11,567 | 323,009 | | 358.1 (358.0 - 358.2) |
| **2003** | 31 | 932 | 332.6 (331.4 - 333.8) | 1,181 | 32,180 | 367.0 (366.8 - 367.2) | 11,140 | 319,392 | | 348.8 (348.7 - 348.9) |
| **2004** | 41 | 927 | 442.3 (440.9 - 443.7) | 1,304 | 34,421 | 378.8 (378.6 - 379.0) | 11,397 | 315,116 | | 361.7 (361.6 - 361.8) |
| **2005** | 42 | 946 | 444.0 (442.7 - 445.3) | 1,384 | 36,505 | 379.1 (378.9 - 379.3) | 11,182 | 312,896 | | 357.4 (357.3 - 357.5) |
| **2006** | 48 | 965 | 497.4 (496.0 - 498.8) | 1,401 | 38,866 | 360.5 (360.3 - 360.7) | 11,064 | 312,100 | | 354.5 (354.4 - 354.6) |
| **2007** | 51 | 977 | 522.0 (520.6 - 523.4) | 1,427 | 41,500 | 343.9 (343.7 - 344.1) | 10,532 | 312,992 | | 336.5 (336.4 - 336.6) |
| **2008** | 52 | 1,025 | 507.3 (505.9 - 508.7) | 1,421 | 44,635 | 318.4 (318.2 - 318.6) | 10,584 | 315,863 | | 335.1 (335.0 - 335.2) |
| **2009** | 47 | 1,041 | 451.5 (450.2 - 452.8) | 1,540 | 47,691 | 322.9 (322.7 - 323.1) | 10,854 | 319,840 | | 339.4 (339.3 - 339.5) |
| **2010** | 52 | 1,043 | 498.6 (497.2 - 500.0) | 1,695 | 51,303 | 330.4 (330.2 - 330.6) | 12,136 | 324,162 | | 374.4 (374.3 - 374.5) |
| **2011** | 61 | 1,049 | 581.5 (580.0 - 583.0) | 1,719 | 55,942 | 307.3 (307.2 - 307.4) | 11,662 | 329,488 | | 353.9 (353.8 - 354.0) |
| **2012** | 51 | 1,066 | 478.4 (477.1 - 479.7) | 1,908 | 60,793 | 313.9 (313.8 - 314.0) | 11,216 | 335,997 | | 333.8 (333.7 - 333.9) |
| **2013** | 53 | 1,118 | 474.1 (472.8 - 475.4) | 1,910 | 64,844 | 294.6 (294.5 - 294.7) | 12,029 | 349,121 | | 344.6 (344.5 - 344.7) |
| **2014** | 70 | 1,190 | 588.2 (586.8 - 589.6) | 2,142 | 68,644 | 312.0 (311.9 - 312.1) | 12,637 | 365,697 | | 345.6 (345.5 - 345.7) |
| **2015** | 48 | 1,256 | 382.2 (381.1 - 383.3) | 2,228 | 73,228 | 304.3 (304.2 - 304.4) | 13,087 | 385,356 | | 339.6 (339.5 - 339.7) |
| **2016** | 61 | 1,326 | 460.0 (458.8 - 461.2) | 2,294 | 78,332 | 292.9 (292.8 - 293.0) | 13,513 | 406,658 | | 332.3 (332.2 - 332.4) |
| **2017** | 55 | 1,411 | 389.8 (369.5 - 390.8) | 2,295 | 82,679 | 277.6 (277.5 - 277.7) | 14,115 | 429,065 | | 329.0 (328.9 - 329.1) |

**Table S9. Incidence rates (IR) of fractures at 10,000 person years (PY) among patients with type 1 diabetes mellitus, type 2 diabetes mellitus, and without diabetes, aged 80+ years, with 95% confidence intervals (CI).**

|  | **80 +** | | | | | | | | | |
| --- | --- | --- | --- | --- | --- | --- | --- | --- | --- | --- |
|  | **Type 1 diabetes** | | | **Type 2 diabetes** | | | **Without diabetes** | | | |
|  | **N** | **PY at risk** | **IR at 10,000 PY (CI)** | **N** | **PY at risk** | **IR at 10,000 PY (CI)** | **N** | **PY at risk** | **IR at 10,000 PY (CI)** | |
| **1997** | 38 | 516 | 736.4 (734.1 - 738.7) | 978 | 15,253 | 641.2 (640.8 - 641.6) | 14,893 | 189,929 | | 784.1 (784.0 - 784.2) |
| **1998** | 28 | 490 | 571.4 (569.3 - 573.5) | 1,053 | 16,054 | 655.9 (655.5 - 656.3) | 14,607 | 189,884 | | 769.3 (769.2 - 769.4) |
| **1999** | 35 | 468 | 747.9 (745.4 - 750.4) | 1,188 | 16,866 | 704.4 (704.0 - 704.8) | 14,934 | 191,420 | | 780.2 (780.1 - 780.3) |
| **2000** | 38 | 463 | 820.7 (818.1 - 823.3) | 1,198 | 17,899 | 669.3 (668.9 - 669.7) | 15,386 | 190,475 | | 807.8 (807.7 - 807.9) |
| **2001** | 36 | 455 | 791.2 (788.6 - 793.8) | 1,236 | 19,084 | 647.7 (647.3 - 648.1) | 15,616 | 194,028 | | 804.8 (804.7 - 804.9) |
| **2002** | 44 | 452 | 973.5 (970.6 - 976.4) | 1,377 | 20,035 | 687.3 (686.9 - 687.7) | 15,896 | 196,095 | | 810.6 (810.5 - 810.7) |
| **2003** | 37 | 463 | 799.1 (796.5 - 801.7) | 1,299 | 20,983 | 619.1 (618.8 - 619.4) | 15,866 | 195,543 | | 811.4 (811.3 - 811.5) |
| **2004** | 36 | 501 | 718.6 (716.3 - 720.9) | 1,401 | 22,078 | 634.6 (634.3 - 634.9) | 15,667 | 195,786 | | 800.2 (800.1 - 800.3) |
| **2005** | 36 | 532 | 676.7 (674.5 - 678.9) | 1,491 | 23,482 | 635.0 (634.7 - 635.3) | 15,682 | 196,857 | | 796.6 (796.5 - 796.7) |
| **2006** | 50 | 533 | 938.1 (935.5 - 940.7) | 1,548 | 24,692 | 626.9 (626.6 - 627.2) | 15,569 | 197,604 | | 787.9 (787.8 - 788.0) |
| **2007** | 38 | 541 | 702.4 (700.2 - 704.6) | 1,663 | 26,011 | 639.3 (639.0 - 639.6) | 15,653 | 197,959 | | 790.7 (790.6 - 790.8) |
| **2008** | 50 | 547 | 914.1 (911.6 - 916.6) | 1,625 | 27,465 | 591.7 (591.4 - 592.0) | 15,528 | 197,028 | | 788.1 (788.0 - 788.2) |
| **2009** | 36 | 558 | 645.2 (643.1 - 647.3) | 1,757 | 29,095 | 603.9 (603.6 - 604.2) | 15,270 | 197,203 | | 774.3 (774.2 - 774.4) |
| **2010** | 39 | 555 | 702.7 (700.5 - 704.9) | 1,895 | 30,729 | 616.7 (616.4 - 617.0) | 15,886 | 196,226 | | 809.6 (809.5 - 809.7) |
| **2011** | 56 | 554 | 1,010.8 (1,008.2 – 1,013.4) | 1,927 | 32,735 | 588.7 (588.4 - 589.0) | 15,494 | 195,216 | | 793.7 (793.6 - 793.8) |
| **2012** | 28 | 559 | 500.9 (499.0 - 502.8) | 1,909 | 34,844 | 547.9 (547.7 - 548.1) | 14,875 | 194,960 | | 763.0 (762.9 - 763.1) |
| **2013** | 48 | 573 | 837.7 (835.3 - 840.1) | 2,103 | 36,584 | 574.8 (574.6 - 575.0) | 15,448 | 195,526 | | 790.1 (790.0 - 790.2) |
| **2014** | 54 | 562 | 960.9 (958.3 - 963.5) | 2,212 | 38,280 | 577.8 (577.6 - 578.0) | 15,409 | 196,049 | | 786.0 (785.9 - 786.1) |
| **2015** | 27 | 552 | 489.1 (487.3 - 490.9) | 2,366 | 40,269 | 587.5 (587.3 - 587.7) | 15,658 | 198,588 | | 788.5 (788.4 - 788.6) |
| **2016** | 48 | 590 | 813.6 (811.3 - 815.9) | 2,356 | 42,245 | 557.7 (557.5 - 557.9) | 15,508 | 200,923 | | 771.8 (771.7 - 771.9) |
| **2017** | 56 | 643 | 870.9 (868.6 - 873.2) | 2,357 | 44,066 | 534.9 (534.7 - 535.1) | 14,915 | 205,012 | | 727.5 (727.4 - 727.6) |
